# Protective effect of IAXO-102 on renal ischemia-reperfusion injury in rats

**DOI:** 10.25122/jml-2022-0280

**Published:** 2023-04

**Authors:** Yahiya Ibrahim Yahiya, Najah Rayish Hadi, Ahmed Abu Raghif, Noor Ghaffar Said AL Habooby

**Affiliations:** 1 Department of Pharmacology, Faculty of Pharmacy, University of Alkafeel, Najaf, Iraq; 2 Department of Pharmacology and Therapeutics, Faculty of Medicine, University of Kufa, Kufa, Iraq; 3 Deptartment of Pharmacology, College of Medicine, Al Nahrain University, Baghdad, Iraq; 4 Medical College, University of Kufa, Kufa, Iraq

**Keywords:** IAXO-102, ischemic stroke (IS), IL-1β, IL-6, TNFα, Bcl-2 HSP27, ischemia-reperfusion injury, IL-1 – Interleukin-1, IL-6 – Interleukin-6, IRI – Ischemia/Reperfusion Injury, TNF-α – Tumor Necrosis Factor alpha

## Abstract

Ischemia/reperfusion injury (IRI) is a common cause of kidney damage, characterized by oxidative stress and inflammation. In this study, we investigated the potential protective effects of IAXO-102, a chemical compound, on experimentally induced IRI in male rats. The bilateral renal IRI model was used, with 24 adult male rats randomly divided into four groups (N=6): sham group (laparotomy without IRI induction), control group (laparotomy plus bilateral IRI for 30 minutes followed by 2 hours of reperfusion), vehicle group (same as control but pre-injected with the vehicle), and treatment group (similar to control but pre-injected with IAXO-102). We measured several biomarkers involved in IRI pathophysiology using enzyme-linked immunosorbent assay (ELISA), including High mobility group box1 (HMGB1), nuclear factor kappa b-p65 (NF-κB p65), interleukin beta-1 (IL-1β), interleukin-6 (IL-6), tumor necrosis factor-α (TNF-α), 8-isoprostane, Bcl-2 associated X protein (BAX), heat shock protein 27 (HSP27), and Bcl-2. Statistical analysis was performed using one-way ANOVA and Tukey post hoc tests. Our results showed that IAXO-102 significantly improved kidney function, reduced histological alterations, and decreased the inflammatory response (IL-1, IL-6, and TNF) caused by IRI. IAXO-102 also decreased apoptosis by reducing pro-apoptotic Bax and increasing anti-apoptotic Bcl-2 without impacting HSP27. In conclusion, our findings suggest that IAXO-102 had a significant protective effect against IRI damage in the kidneys.

## INTRODUCTION

Ischemia-reperfusion injury (IRI) is a significant contributor to chronic kidney disease (CKD) and related mortality. Hypotension, sepsis, and surgical protocols [1,2] are among contributing factors. Pathological mechanisms of IRI include inflammation and oxidative stress. The prevalence of chronic kidney disease in recent years has led to a large number of kidney transplants being performed [3-5]. Multifunctional compounds with antioxidant, anti-inflammatory, and anti-inflammatory capabilities serve as the best defenses in cases of kidney damage. The protective properties of antioxidants can be explained by their ability to restore intracellular processes related to oxidative damage kinetics [6,7]. In addition, antioxidant therapy can protect against the oxidative damage caused by infrared radiation. Antioxidant compounds have been found to restore intracellular processes related to oxidative damage, potentially contributing to their protective properties [8-11]. Inflammation caused by ischemia-reperfusion injury (IRI) can lead to further renal damage, but protecting against this occurrence is possible [12,13]. Pro-inflammatory cytokines, such as tumor necrosis factor (TNF-), interleukin-1 (IL-1), and interleukin-6 (IL-6), play a primary role in renal disease [13-16]. Moreover, IRI is less common in TLR4 knockout mice or kidneys from donors with TLR4 loss of function, which is associated with a lower concentration of pro-inflammatory cytokines in the kidney and better immediate graft function [17-21]. This study aimed to investigate the nephron-protective effects of IAXO-102 on renal ischemia-reperfusion injury in rats by measuring the following parameters: neutrophil gelatinase-associated lipocalin (NGAL), nuclear factor kappa B-p65 (NF-κB), interleukin-1 beta (IL-1β), B-cell lymphoma-2-associated X protein (BAX), interleukin-6 (IL-6), tumor necrosis factor-alpha (TNF-α), high mobility group box 1 protein (HMGB-1), B-cell lymphoma 2 protein (Bcl-2), heat shock protein 27 (HSP27), and 8-isoprostane.

## Material and Methods

### Animals

In this study, 24 adult male Wistar Albino rats aged more than 20 weeks and an average weight of 300±50 g were used. All animals had free access to food and water and were subjected to a 12:12 light-dark cycle. The temperature and humidity were controlled at 25 °C and 60-65%, respectively. The rat handling, experiments, and tests complied with the Ethical Conduct for Use of Animals guidelines and regulations. The animals were housed at the animal house of the College of Sciences, University of Kufa. The materials were procured from Merck Chemical Companies (Kufa University).

### Experimental design

In this study, Wistar Albino rats were randomly selected and divided into four groups, each consisting of six rats, subjected to different handling procedures. The sham group served as the negative control and underwent no IRI procedure. The control group underwent bilateral renal ischemia by clamping the renal pedicles for 30 minutes, serving as the positive control. The vehicle-treated group received an intraperitoneal injection of 10% ethanol, 40% PEG300, 5% tween-80, and 45% saline (the vehicle for IAXO-102) one hour prior to the production of bilateral renal ischemia-reperfusion damage. Rats in the last group were intraperitoneally administered 3 mg/kg of IAXO-102 an hour before establishing bilateral ischemia-reperfusion damage.

### Sample collection and preparation

#### Serum tests

After the reperfusion period, blood was collected via cardiac puncture from each experimental rat using disposable syringes. Approximately 3-5 ml of blood was collected and transferred to gel tubes without anticoagulant, then left at 37°C for 20 minutes. After centrifuging the tubes at 3000 rpm for 10 minutes, the serum was collected [22]. The serum samples were then divided into aliquots, one of which was used to measure serum urea, creatinine, and neutrophil gelatinase-associated lipocalin (NGAL) levels.

#### Renal tissue examination

Parts of the left kidneys were fixed in 10% neutral buffered formalin from sham, control, vehicle, and drug-treated rats. The fixed tissues were processed using an automated Leica tissue processor, which dehydrates the tissues with serially increasing ethanol concentrations, clears the tissues with xylene to form ethanol and some fats that impede wax infiltration, and finally prepares paraffin-embedded tissue blocks. These blocks were then cut into tissue sections with a thickness of about 4 micrometers using a microtome. The tissue sections were then mounted on slides and later stained with H&E dyes to assess renal tissue damage.

### Statistical analysis

The Statistical Analysis System (SAS) version 9.1 was used for data analysis. To determine significant differences in ELISA results, one-way ANOVA and the Tukey post hoc test were employed. Post hoc tests are crucial in ANOVA analysis. In creating the figures, SPSS version 23 was used. A p-value of less than 0.05 was considered statistically significant.

## Results

### Effect of IAXO-102

**Figure 1:**
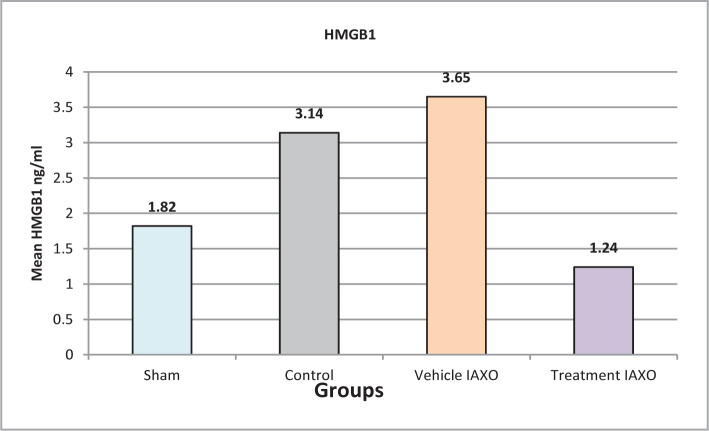
Mean serum levels of HMGB1

In this study, four groups, each consisting of six rats, were enrolled to compare different parameters among the studied groups and assess the effects of IRI and IAXO-102 on inflammatory mediators and HMGB1. There was a significant difference in HMGB1 levels among the four studied groups. However, the most important finding was that the IAXO-102 group had significantly lower HMGB1 levels than all other groups (P<0.05), as illustrated in Figure 1.

### Effect of IRI, IAXO-102 on NFκp65

**Figure 2:**
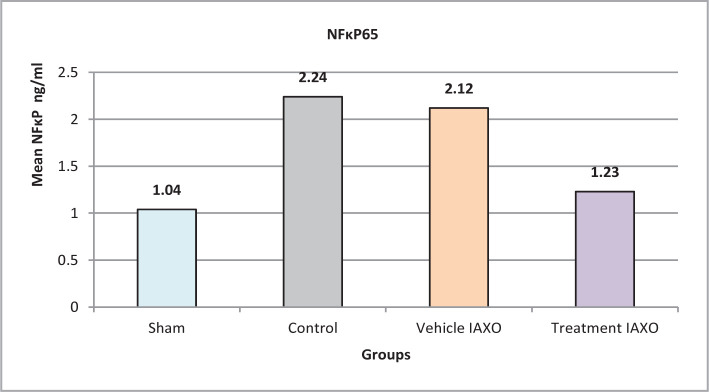
Mean serum levels of NFκp65.

The mean NFκp65 levels were significantly lower in the IAXO-102 group compared to all other groups (P<0.05), except for the sham group (P>0.05) (Figure 2).

### Effect of IRI, IAXO-102 on IL-1β

**Figure 3:**
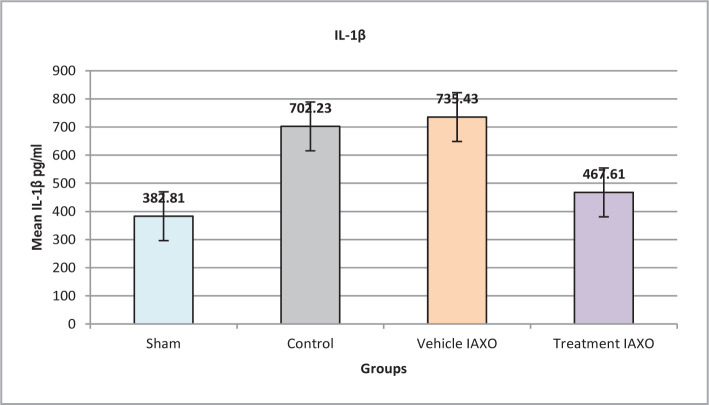
Mean serum levels of IL-1β.

There was a significant difference in IL-1β2 levels among the studied groups (P<0.001). The sham group had the lowest levels, significantly lower than all other groups (P<0.05 for all comparisons). On the other hand, IL-1β levels were significantly lower in the treatment IAXO, vehicle IAXO, and control groups (P<0.001) (Figure 3).

### Effect of IRI, IAXO-102 on IL-6

**Figure 4:**
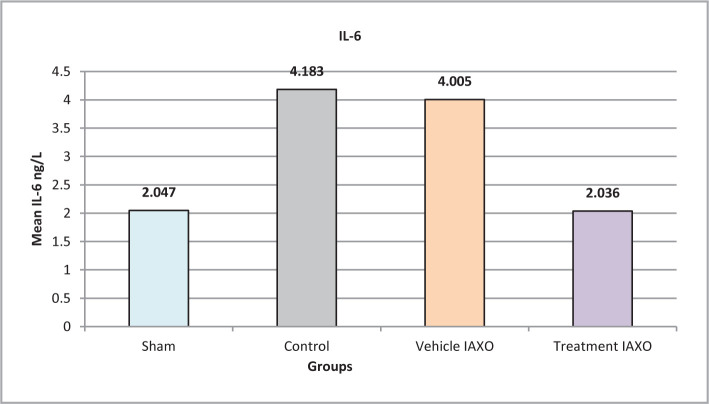
Mean serum levels of IL-6.

The mean IL-6 levels in the sham group were significantly lower compared to the control and vehicle IAXO groups, whereas the treatment IAXO group had a significantly higher level of IL-6 than all other groups (P<0.05). IL-6 levels were significantly lower than those in the control and the vehicle IAXO groups (P=0.001), except for the treatment IAXO group, which had an elevated IL-6 level (P<0.05), as shown in Figure 4.

### Effect of IRI, IAXO-102 on TNF-α

**Figure 5:**
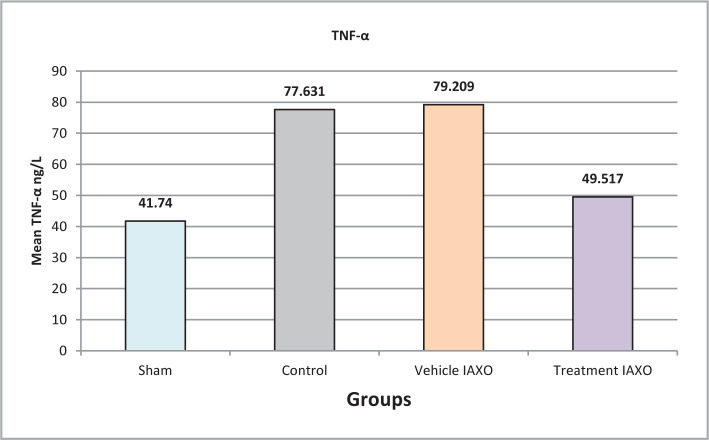
Mean serum levels of TNF-α.

The mean TNF-α levels were significantly lower in the IAXO-102 group than in all other groups (P<0.05), except for the sham group (P>0.05) (Figure 5).

### Effects of renal IRI, IAXO-102 on the oxidative stress marker 8-isoprostane

**Figure 6:**
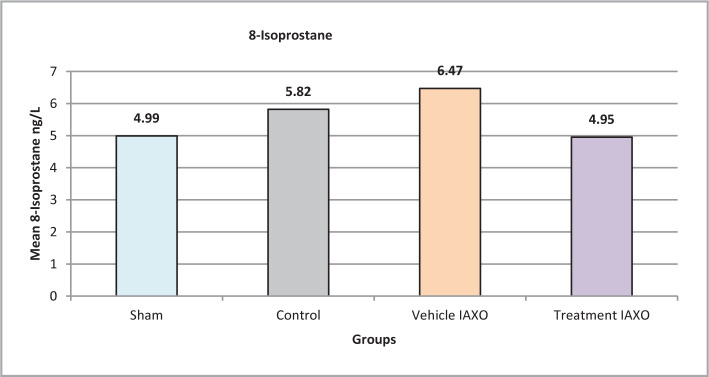
Mean serum levels of 8-Isoprostane.

There was a significant overall difference in the mean 8-isoprostane levels among the groups (P=0.001), as shown in Figure 6. The control group had significantly lower levels of 8-isoprostane compared to the other groups, and the treatment IAXO group also had significantly lower levels than the vehicle IAXO group (P<0.05).

### Effects of renal IRI, IAXO-102 on apoptotic mediators

#### Effect of renal IRI, IAXO-102 on Bax

**Figure 7:**
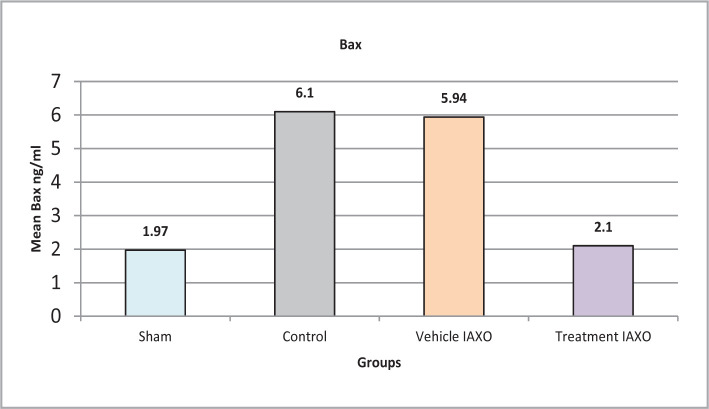
Mean serum levels of Bax

Significant differences were observed in the mean Bax levels among the four groups (P value <0.001), as shown in Figure 7. The Sham group had the lowest level of Bax, while the control group and Vehicle IAXO had higher levels. The treatment IAXO group had a significantly lower level of Bax compared to the control and Vehicle IAXO groups (P<0.05).

#### Effect of renal IRI, IAXO-102 on HSP27

**Figure 8:**
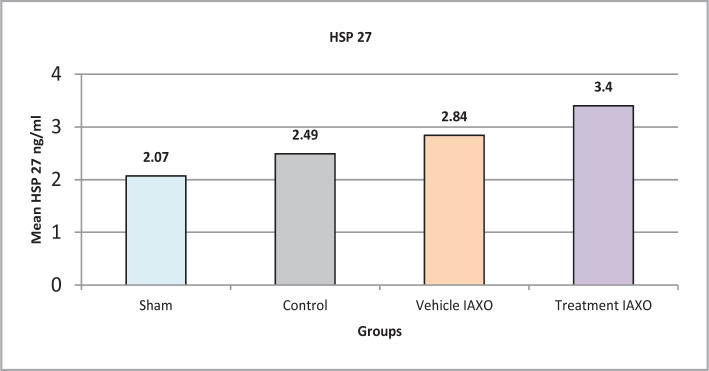
Mean serum levels of HSP27.

The mean level of HSP27 was significantly lower in the sham group than in all other groups except for the control group In contrast, the IAXO-102 group had a significantly higher level of HSP27 than the control group (P<0.05) but not significantly different from the other groups (Figure 8).

#### Effect of renal IRI, IAXO-102 on Bcl-2

**Figure 9:**
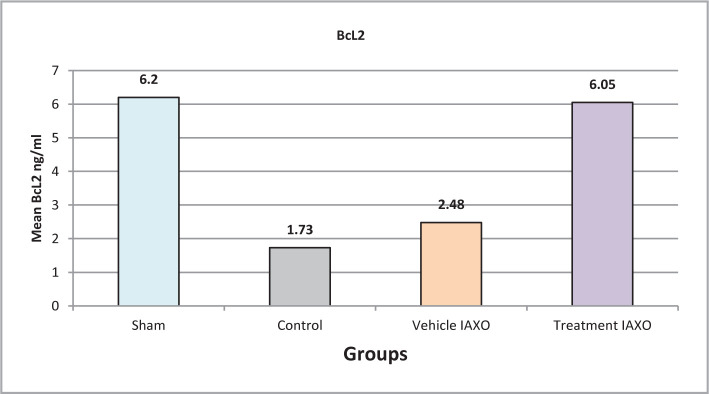
Mean serum levels of BcL2.

The mean levels of Bcl-2 were significantly higher in the treatment group than in all other groups, except for the sham group (P<0.05), as shown in Figure 9.

## Discussion

The reperfusion process following ischemic injury can exacerbate tissue damage by activating innate immune responses and cell death pathways [23]. This study aimed to assess the effect of IAXO-102 on renal ischemia-reperfusion injury using a rat model. We analyzed various experimental studies simulating ischemia-reperfusion (I/R) kidney damage in laboratory animals.

### Effects of renal IRI, IAXO-102 on inflammatory mediators

#### Effect of renal IRI, IAXO-102 on HMGB1

HMGB1 is known to play a crucial role in the early stages of I/R injury by binding to RAGE and triggering the activation of pro-inflammatory pathways, leading to increased ischemia injury. Thus, blocking HMGB1 may provide a potential therapeutic strategy for I/R injury [24]. In this experimental study, we compared the levels of HMGB1 in different groups and found that pretreatment with IAXO-102 significantly reduced the level of HMGB1, suggesting that IAXO-102 could be used as a standalone therapy for I/R injury.

#### Effect of renal IRI, IAXO-102 on NF-κB

The mean NF-κB levels were significantly reduced in the pretreatment group, IAXO-102. It is widely recognized that the inhibition of the NF-κB pathway has the potential to protect against ischemia/reperfusion injury, although the exact mechanism of action is not fully understood [25,26]. Nonetheless, the expression of the inflammatory gene, including IL-1β, IL-6, IL-10, and TNF-α, in addition to adhesion factors, can be promoted by NF-κB activation [26]. The increased expression of adhesion molecules attracts more neutrophils and lymphocytes, leading to further injuries to vascular endothelium cells. Therefore, the use of an anti-inflammatory agent may have a promising protective effect on renal IRI, as demonstrated by the significantly reduced NF-κB levels observed in all three treatment modalities in our study [25].

#### Effect of IAXO-102 on IL-1β2, IL-6 and TNFα

Previous experimental studies have demonstrated that the levels of IL-12 and TNF-α increase after 30 minutes of ischemia followed by 2 hours of reperfusion in rats, resulting in significant changes in endothelial function and contributing to endothelial dysfunction. In the present study, IL-12 and TNF levels were significantly lower in the treatment group and elevated in the vehicle IAXO, control, and sham groups after IRI. [26,27]. Another study in a rat model showed that the TNF-α gene level was highly elevated in injured kidney tissues in rats that underwent a right kidney nephrectomy and ischemia in the left kidney for 45 minutes, followed by reperfusion [28]. Moreover, two other experimental studies on a rat model showed that the IL-1β level was increased in injured renal tissues after 30 minutes of ischemia, followed by 2 hours of reperfusion [29,30]. Based on the experimental studies, it has been shown that spinal cord injuries lead to the initiation of local inflammatory processes and the formation of inflammatory cytokines such as TNFα and IL-1β. In contrast, other studies have reported that upregulation of nuclear factor erythroid 2-related factor (Nrf2/HO-1) can promote a protective effect against hepatotoxicity caused by cyclophosphamide through the attenuation of oxidative stress, inflammation, and cell death signaling [31].

In the current study, pretreatment with IAXO-102 significantly reduced IL-6 levels compared to the control and vehicle IAXO groups (P<0.001). These findings correspond with previous studies that reported IL-1 as a stimulus for producing pro-inflammatory cytokines such as TNF-alpha and IL-6. Furthermore, the C5a component of the complement system was found to activate the production of MCP-1 chemokine, IL-1, TNF-alpha, and IL-6 cytokines. IL-1 also induces the expression of adhesion molecules on endothelial cells, promoting cell infiltration, and stimulates the production of prostaglandins and other inflammatory mediators, such as TNF-alpha and IL-6, by tubular epithelial cells. Therefore, IAXO-102 may act through an anti-inflammatory mechanism to reduce or prevent IRI damage by inhibiting the production of IL-6 and other pro-inflammatory cytokines [32,33].

### Effect of IRI, IAXO-102 on the oxidative stress marker 8-isoprostane

In this experimental rat model study, we reported a statistically significant overall difference in the mean 8-Isoprostane levels among the groups (P=0.001). The treatment IAXO group had significantly lower levels of 8-isoprostane, so we can hypothesize that IAXO can improve the outcome after ischemia/reperfusion injury and reduce its adverse effect. Previous studies have demonstrated that during IRI, the burst of reactive oxygen species (ROS) can trigger inflammation and tubular cell injury. Therefore, reducing oxidative stress, as assessed by 8-isoprostane, may help prevent damage in IRI [34]. Moreover, data in other experimental studies demonstrate that IAXO-102 treatment is a negative regulator of Angiotensin II-driven inflammation, and TLR4 signaling is the target by which IAXO-102 exerts its effect and attenuates inflammation [35,36].

### Effect of IRI, IAXO-102 on the Apoptotic mediators

#### Effect of IAXO-102 on Bcl-2 and Bax

Pretreatment with IAXO-102 in our experimental study significantly reduced the levels of Bcl-2 compared to other groups. These findings are consistent with previous research that demonstrated a significant increase in Bax levels and a decrease in Bcl-2 levels in the control group compared to the sham group following 30 minutes of renal ischemia and 72 hours of reperfusion [37,38]. Bax and Bcl-2 are known to play crucial roles in regulating apoptosis, even though additional Bcl-2 members most likely play "supportive functions" in cell death. Additionally, targeting either or both of these proteins can modify the outcome of stress-induced cell death [39].

#### Effect IRI, IAXO-102 on HSP

IAXO-102 pretreatment significantly increased the levels of heat shock protein compared to the other groups, although statistical significance was not reached in the other groups, possibly due to the limited sample size. Heat shock proteins (HSPs) have been shown to have potent anti-apoptotic effects, blocking various steps in the cell death pathway and inducing cytoprotection in vitro or in vivo [39]. Therefore, the use of IAXO-102 for pretreatment may be critical in this mechanism. Other research has shown that essential chemicals involved in cell survival and proliferation are upregulated, and miRNA expression in I/R alters apoptosis (e.g., Bcl-2, HSP). miRNA expression profiling has been used to identify differential regulation of numerous miRNAs in several organs after I/R to modulate genes involved in cell death. HSP also prevents Bax from activating [40].

## Conclusion

The study found that IAXO-102 has a protective effect against IRI damage, suggesting its potential as a therapeutic intervention.
